# Directed Experimental Adaptive Evolution of Osmoregulation in Fungal Pathogen *Magnaporthe oryzae* Is Independent of Glycerol Metabolism-Associated Genes

**DOI:** 10.3390/biology14111545

**Published:** 2025-11-04

**Authors:** Katharina Bersching, Christiane Grünewald, Stefan Jacob

**Affiliations:** 1 Institute of Biotechnology and Drug Research gGmbH (IBWF), Hanns-Dieter-Hüsch-Weg 17, D-55128 Mainz, Germany; 2 Microbiology and Biotechnology at the Institute of Molecular Physiology, Johannes Gutenberg-University Mainz, Hanns-Dieter-Hüsch-Weg 17, D-55128 Mainz, Germany

**Keywords:** directed experimental adaptive evolution, high osmolarity glycerol (HOG) pathway, *Magnaporthe oryzae*, osmoregulation, suppressor

## Abstract

Directed experimental adaptive evolution in fungal pathogens is largely unexplored. In the phytopathogenic fungus *Magnaporthe oryzae*, long-term cultivation under osmotic stress was found to lead to individuals arising as suppressor strains out of osmosensitive “loss-of-function” mutants, in which the high osmolarity glycerol (HOG) pathway was inactivated. The underlying mechanisms of reestablished osmoregulation in the suppressor strains are not known. Here, we found that two different types emerged from the mycelium parts of each *∆Mohik1*, *∆Moypd1*, *∆Mossk1*, *∆Mossk2*, *∆Mopbs2,* and *∆Mohog1*: reversible suppressors, which still struggle with osmotic stress, and irreversible suppressors, which can cope with the same stress situations. This phenomenon only takes place in lof mutants, which are related to the HOG pathway and are not in other osmosensitive mutants. Both suppressor types produce glycerol as a stress response instead of arabitol as it is in the wildtype strain. Glycerol production was found to be almost twice as high in the irreversible strains as compared to the reversible strains. Thus, glycerol metabolism (gm) was assumed to be involved in the molecular mechanism of this adaptive-driven evolution. We generated a set of double mutant strains in which we deleted different gm-related genes within the HOG lof-mutants. Since suppressors originate from these double lof-mutants upon long-term stress, we exclude gm-associated genes acting as drivers for adaptive-driven evolution.

## Simple Summary

The world’s population is rising rapidly, and a major problem is global food security. *Magnaporthe oryzae* is placed first on a list of the world’s top ten plant pathogens with the highest scientific and economic importance since it causes blast, which is the most devastating disease of cultivated rice—the major food source for more than half of the world’s population. In this study, we demonstrate directed experimental adaptive evolution in this fungus and give insights into this evolutionary phenomenon with regard to the molecular mechanisms behind it. These results will help us better understand the molecular basis of evolutionary events of the rice blast fungus and may open the door to developing new strategies for plant protection and food security in the future. In addition, our study is a valuable contribution to the basic research area of signaling mechanisms in filamentous pathogenic fungi and will thus be of considerable interest to a broad readership within the scientific community.

## 1. Introduction

Evolution is generally understood as a slow process acting over thousands of years, but in recent decades, research in the field of evolutionary dynamics or directed experimental adaptive (DEA) evolution has shed light on biological processes in microorganisms, which are found to be much faster [1]. Yet, the molecular mechanisms causing these rapid evolutionary adaptations are not well understood because they rely on rarely studied, often unpredictable, spontaneous mutations. Research in this area is not only of academic significance but also of critical importance for understanding how organisms handle global challenges, such as changes in ecosystems, climate change, the emergence of pathogens, the expansion of invasive organisms, and, of course, the (multi-) resistance to vaccines and drugs [2]. However, rapid adaptation of microorganisms to changing environmental conditions can take place even within a few generations, facilitating observations of adaptations, genetic evolution of populations, and competitive dynamics [3]. The majority of research efforts in this field so far have focused on bacterial systems [4]. Evolutionary adaptation of microorganisms is often explained by the instability of chromosomes after duplication of the whole genome [5]. Furthermore, transposable elements are discussed intensively as driving forces in the evolution of organisms [6]. Both of these examples lead to a diversification of species by restructuring of the genome. In one of the world’s top ten most dangerous pathogens in crops, the rice blast fungus *Magnaporthe oryzae* (syn. *Pyricularia oryzae*), which is the underlying mechanism for “normal” evolution, are documented so far in high variation in nucleotides, high substitution ratios, and frequent polymorphisms [7]. Pathogenic organisms like *M. oryzae* are forced to constantly adapt to changing environmental stimuli and dynamic changes during host–pathogen interactions [8]. In addition to “long-term” adaptation mediated by alterations in the genome or epigenetic changes, the facultative pathogenic lifestyle requires a rapid adaptive behavior, which enables a fast regulation of cellular processes. For this, a dynamic alteration of protein structure or protein–protein interaction is facilitated by post-translational modifications (PTMs) that are both known to be highly flexible and partially reversible [9]. The genome of *M. oryzae* encodes a huge set of protein kinases and more than 60 peptidases, highlighting the biological importance of such PTMs. To date, almost nothing is known about evolutionary dynamics and the molecular mechanisms behind the modulation of signaling networks regulating physiological and biochemical systems/processes [10]. Signaling pathways cannot be studied as single modules, but rather are seen as complex networks of interactions, often including several different pathways, transcriptional regulations, and biochemical mechanisms [11]. That complicates the search for single evolutionary events because a small change in just one molecule can affect an entire network of signaling pathways [12]. In this context DEA represents a powerful methodology within evolutionary biology, enabling researchers to observe and navigate the evolutionary way of microorganisms under controlled laboratory conditions. Through DEA, microbial populations are subjected to specific environmental stresses or selective pressures over multiple generations, allowing for the rapid accumulation of beneficial mutations and the emergence of adaptive traits. By iteratively exposing organisms to challenging conditions and selecting for desired phenotypes, DEA facilitates the study of evolutionary dynamics in real time, shedding light on the underlying genetic and physiological mechanisms driving adaptation. This approach not only provides valuable insights into the evolutionary processes occurring within microbial populations but also offers practical applications in diverse fields such as biotechnology, medicine, and environmental science.

In contrast to most of the classical metabolic pathways, many signal transduction pathways use several modular mechanisms to route and coordinate an input–output interaction [11]. One prominent example is the high osmolarity glycerol (HOG) pathway in the fungal pathogen *Magnaporthe oryzae* [13]. The physiological role of the HOG pathway is to regulate the adaptation of the cells to increased osmolarity in the surrounding environment. The molecular architecture of the HOG pathway consists of a phosphorelay system composed of MoSln1p/MoHik1p-MoYpd1p-MoSsk1p and a downstream MAPK cascade (MoSsk2p-MoPbs2p-MoHog1p) [14]. Targets of MAPK generally include plenty of receivers like transcription factors, phosphatases, further MAPK-activated protein kinases, and other protein classes that regulate physiological processes, cell morphology, cell cycle progression, metabolism, and gene expression in response to various extracellular signals or environmental stresses [15]. The HOG pathway in *M. oryzae* has already proven to be a suitable model system for fundamental research of physiological functions and the mode of action of agricultural fungicides (e.g., fludioxonil) [14]. Initially, loss-of-function (lof) mutants of the HOG pathway were used to characterize the individual functional elements [16]. It is known that these lof mutants are not only osmosensitive, but also fail to produce important compatible solutes such as arabitol to cope with osmotic stress. The sugar alcohol arabitol is produced by the *M. oryzae* wildtype strain in response to osmotic stress [17]. In 2019, Bohnert et al. cultivated the osmosensitive lof mutants *ΔMohog1*, *ΔMopbs2*, *ΔMossk2*, *ΔMossk1*, and *ΔMoypd1* for several weeks under permanent osmotic stress and found stable individuals arising from the mycelium of each lof mutant. Interestingly, these rapidly “evolved” suppressor strains are not only able to handle osmotic stress again, but produce glycerol instead of arabitol in response to osmotic stress [14,18]. In line with this, it was hypothesized in a previous study that glycerol metabolism-dependent genes may be responsible for the suppressor phenotype [18]. A set of candidate genes was found to be upregulated in both the salt stress samples of the *ΔMohog1(adapted)* and the wildtype strain, whereas these genes were not regulated in the lof mutant *ΔMohog1*. Among these candidates were the glycerol H+-symporter MoSlt1p (MGG_09852), the phosphoglycerate mutase (MGG_06642), one glycerol-3-phosphate dehydrogenase (MGG_00067 (MoGpd1p)), and one phosphatidyl synthase (MGG_00099 (MoHad1p)).

In contrast to previous hypotheses, the inactivation of these candidate genes within the lof HOG mutants does not result in the absence of the DEA suppressor phenotype, and glycerol metabolism-dependent genes can be excluded from being involved in DEA of *M. oryzae*. As a consequence, glycerol production within the suppressor strains and the underlying molecular mechanisms of DEA are still to be discovered, which underscores the complexity of the evolutionary mechanisms of signaling networks in *M. oryzae*.

## 2. Materials and Methods

### 2.1. Cultivation of Magnaporthe oryzae

The *Magnaporthe oryzae* strains used in this study were *M. oryzae* 70-15 (MoWT, Fungal Genetics Stock Center, Manhattan, KS, USA), the loss-of-function mutants *∆Mohik1* (MGG_11174), *∆MoYpd1* (MGG_07173), *∆Mossk1* (MGG_02897), *∆Mossk2* (MGG_00183), *∆Mopbs2* (MGG_10268), *∆Mohog1* (MGG_01822) [13] and to all the lof mutants the corresponding suppressor strains [18]. The strains were grown at 26 °C on complete medium (CM). The CM at pH 6.5, 2% agar, contains per liter: 1 g casamino acids, 10 g glucose, 2 g peptone, 1 g yeast extract, 50 mL nitrate salt solution (containing per liter: 10.4 g KCl, 30.4 g KH_2_PO_4_, 10.4 g MgSO_4_·7H_2_O, 120 g NaNO_3_) and 1 mL of a trace element solution (containing per liter: 1.7 g CoCl_2_·6 H_2_O, 1.6 g CuSO_4_·5H_2_O, 5 g FeSO_4_·7H_2_O, 11 g H_3_BO_3_, 5 g MnCl_2_·4H_2_O, 50 g Na_2_EDTA, 1.5 g Na_2_MoO_4_·2H_2_O, 22 g ZnSO_4_·7H_2_O, pH 6.5 adjusted by 1 M KOH). All chemicals used were p.a. quality unless stated otherwise.

### 2.2. Single Spore Isolation

Conidia of each strain (approximately 11 days old) were filtered through two layers of Miracloth tissue (Merck). The suspension was adjusted to 1 × 10^3^ conidia mL^−1^ in sterilized H_2_O. About 50–100 µL of the conidia suspension was placed on a selection medium and incubated at 26 °C for about 12–24 h. After the incubation, colonies growing from single spores are visible, can be selected and placed on a new medium containing the selection antibiotic for further incubation.

### 2.3. Construct of Vectors for Genetic Manipulation

The gDNA from *Magnaporthe oryzae* was isolated from mycelium of three-day-old liquid cultures [13]. For the purification the GeneJET^TM^ Plant Genomic DNA Purification Mini Kit (Thermo Fisher Scientific, Waltham, MA, USA) was used according to the manual’s user guide. The procedures of standard molecular cloning were based on [19]. All plasmids used in this study were generated by using the Gibson assembly cloning method. As a backbone the BglII/PstI-restricted plasmid psj-basic was used [19]. The exchange of the resistance cassette of the inactivation mutants was conducted via homologous recombination. Therefore, the flanks were chosen before the start codon and after the stop codon. The size of the flanks can be different between the mutants because care was taken not to interrupt or unintentionally modify other genes. The sizes of the flanks can vary between 250 and 3900 bp. Bigger flanks were chosen if genes are located nearby to further avoid unattentional inactivation of them. The flank (flank 1/flank 2) size for the reintegration of the original HOG genes were: *∆Mohik1/HIK1* (1000 bp/1000 bp), *∆Moypd1/YPD1* (600 bp/330 bp), *∆Mossk1/SSK1* (500 bp/1950 bp), *∆Mossk2/SSK2*, *∆Mopbs2/PBS2* (500 bp/3900 bp) and *∆Mohog1/HOG1* (1500 bp/998 bp) and for the inactivation mutants: *∆Mostl1* (700 bp/700 bp), *∆Mogpd1* (700 bp/700 bp), *∆Mopga1* (841 bp/683 bp) and *∆Mohad1* (1000 bp/100 bp). The BAR resistance cassette was amplified from a modified bialaphos resistance gene, BAR [14].

For bacterial transformation NEB^®^ 10-β competent *Escherichia coli* strains (high efficiency) were used. The fungal transformation was performed via Agrobacterium tumefaciens-mediated transformation [20,21,22], resulting in the mutant strains listed in the Supplementary Materials. The selection of transformants containing resistance to glufosinat-ammonium was performed by using 100 µg mL^−1^ of the antibiotic in minimal medium (MM, [13]), and the successful replacement within the gDNA of the mutants was confirmed by Southern blot analysis or sequence-specific PCR screens (Figures S1–S4).

All oligonucleotides used in this study were generated by Eurofins Genomics (Ebersberg, Germany) and listed in the Supplementary Materials.

### 2.4. Plant Infection Assays

The plant infection assays were carried out in order to assess virulence as described previously [23] and performed with MoWT, the lof-mutants of the HOG pathway, the corresponding suppressor strains, and the generated complementation mutants. For the plant infection assay, the conidia of the above-mentioned strains were harvested from 11-day-old cultures, which were cultivated on CM. The cultivated rice plants (CO39) were grown for 21 days using daily cycle of 16 h light and 8 h darkness at 28 °C. The plants were spray inoculated with the conidial suspensions (diluted in H_2_O containing 0.2% gelatin). After 5 days of incubation, lesions were analyzed. Five biological replicates were analyzed.

### 2.5. Vegetative Growth Assays

Agar blocks of approximately 0.8 cm diameter were cut from the cultures and placed onto freshly prepared CM or Minimal medium (pH 6.5, 2% agar, contains per liter: 1 g glucose, 50 mL nitrate salt solution, 0.25 mL biotin-solution (0.01%), 1 mL thiamin dichloride solution (1%) and 1 mL of trace elements) with different compounds to induce stress. The cultures were grown for 10 days at 26 °C.

### 2.6. Germination Assay

The germination assay was carried out according to [16]. The conidia were filtered through two layers of miracloth tissue (Merck, Darmstadt, Germany) to give a conidial suspension, which was adjusted to 5 × 10^4^ conidia mL^−1^ in H_2_O. Then, the samples were incubated at 26 °C for at least 16 h. The germination and the subsequent initial vegetative growth phase were monitored under the microscope.

### 2.7. HPAEC-PAD Analysis to Quantify Compatible Solute Production

The osmolytes (sugar alcohols, monosaccharides, and disaccharides) were detected by the HPAEC-PAD analysis as described previously [24]. Different stressors besides 0.5 M KCl were tested and are listed in the Section 3.

### 2.8. Data Statistics and Analysis

All quantitative experiments were performed with at least three independent biological replicates unless otherwise stated in the figure legends. Raw data were first inspected for normality using the Shapiro-Wilk test and for homogeneity of variances with Levene’s test (implemented in R version 4.4.0).

Parametric data—When both assumptions were met, pair-wise comparisons were carried out with two-tailed Student’s *t*-tests. For experiments involving three or more groups, one-way analysis of variance (ANOVA) was applied, followed by Tukey’s honestly significant difference (HSD) post-hoc test to identify specific group differences.

Non-parametric data—If normality or equal-variance criteria failed, the Mann-Whitney U test (two groups) or the Kruskal–Wallis test with Dunn’s multiple-comparison correction (multiple groups) was used.

Statistical calculations were performed in R and visualized with the ggplot2 package (4.0.0). Figures show the mean ± standard deviation (SD) unless otherwise indicated; for data with skewed distributions, the median and inter-quartile range (IQR) are reported. Statistical significance was accepted at *p* < 0.05. 

## 3. Results

### 3.1. Directed Experimental Adaptive Evolution Results in Two Distinct Types of Suppressors

**Figure 1:**
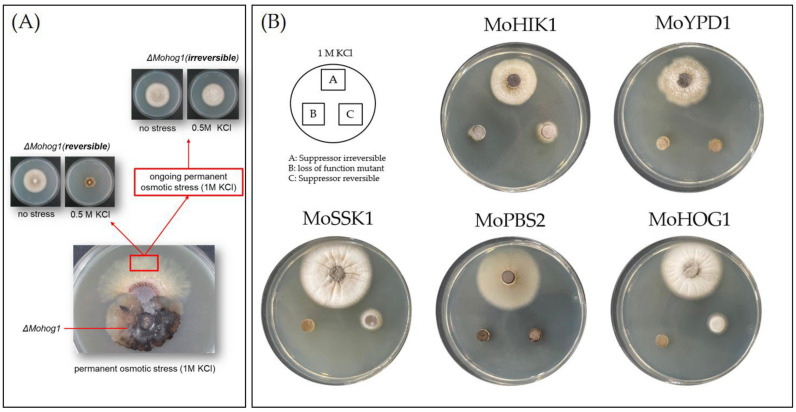
Directed experimental adaptive (DEA) evolution in *Magnaporthe oryzae* upon osmotic stress. (**A**) Two types of DEA: reversible (**left**) and irreversible (**right**) suppressor mutants arise out of lof mutants with an inactivated HOG pathway upon long-term cultivation under osmotic stress. (**B**) Overview of vegetative growth of the *Magnaporthe oryzae* lof-mutants and the suppressor strains (single spore isolates of the reversible and irreversible suppressors) for 5 days upon osmotic stress (1 M KCl).

*Magnaporthe oryzae* lof mutants with an inactivated HOG pathway are sensitive to osmotic stress. In our previous study, it was found that under permanent osmotic stress, individual suppressor strains with reestablished osmoregulation arise out of these lof mutants [18]. In ongoing investigations of these suppressor strains (formerly identified and referred to as “adapted” strains, [18]), which have originated from *∆Mohik1*, *∆Moypd1*, *∆Mossk1*, *∆Mossk2*, *∆Mopbs2*, and *∆Mohog1*, we recently identified at least two types (or two “stages”) of suppressor strains. Therefore, we hypothesized that the mechanism of directed experimental adaptive (DEA) evolution is even more dynamic than originally expected (Figure 1A).

Initially, individual mycelium parts were found outgrowing from the mycelium of each lof mutant *∆Mohik1*, *∆Moypd1*, *∆Mossk1*, *∆Mossk2*, *∆Mopbs2*, and *∆Mohog1* after about three to four weeks of cultivation on permanent osmotic stress. These individual mycelium parts were isolated, named as “reversible”, and subjected to further investigations. After two more weeks of constant stress, additional pieces of mycelium grew out of the original cultures, and these were named “irreversible” (Figure 1A).

After sub-cultivating under unstressed conditions on CM medium for 8–11 days, both suppressor types have been re-cultivated upon osmotic stress (e.g., KCL or Sorbitol). Surprisingly, not all suppressor mutants were able to cope with osmotic stress. We could clearly distinguish two different types of suppressor strains: (I) the reversible suppressor mutants, which are found to be impaired in the ability to cope with repeated osmotic stress; (II) the irreversible suppressor mutants, which are able to grow under osmotic stress (Figure 1A). From each reversible and irreversible suppressor mutant arising out of *∆Mohik1*, *∆Moypd1*, *∆Mossk1*, *∆Mossk2*, *∆Mopbs2*, and *∆Mohog1*, single spore isolation was performed in order to safely separate only pure cultures. The individually strains, which were obtained by single spore isolation, have been named *∆Mohik1*(reversible), *∆Moypd1*(reversible), *∆Mossk1*(reversible), *∆Mossk2*(reversible), *∆Mopbs2*(reversible), *∆Mohog1*(reversible), and *∆Mohik1*(irreversible), *∆Moypd1*(irreversible), *∆Mossk1*(irreversible), *∆Mossk2*(irreversible), *∆Mopbs2*(irreversible) and *∆Mohog1*(irreversible), respectively.

**Figure 2:**
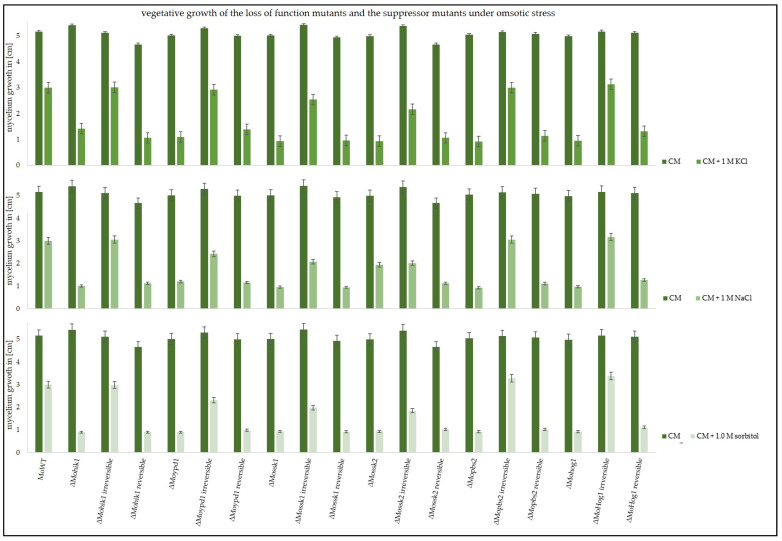
Vegetative growth of the wildtype strain (MoWT), the original lof mutants, and the suppressor strains (irreversible and reversible). Growth diameter of the colonies was measured after 7 days on CM, or CM supplemented with 1 M KCl, 1 M NaCl, or 1 M sorbitol. The experiments were conducted in triplicate.

These suppressor strains were then used in vegetative growth assays in order to compare their ability to cope with osmotic stress. Apart from KCl stress, NaCl as well as sorbitol were used as stress-inducing agents (Figure 2).

All irreversible suppressor strains, ∆Mohik1(irreversible), ∆Moypd1(irreversible), ∆Mossk1(irreversible), ∆Mossk2(irreversible), ∆Mopbs2(irreversible), and ∆Mohog1(irreversible), were found to be almost as vital as the wildtype strain in the different stressed situations. In contrast, the growth of reversible suppressor strains ∆Mohik1(reversible), ∆Moypd1(reversible), ∆Mossk1(reversible), ∆Mossk2(reversible), ∆Mopbs2(reversible), and ∆Mohog1(reversible) was strongly impaired as compared to the irreversible strains and the wildtype strain. But nevertheless, it has to be mentioned that the reversible strains are not as sensitive as the lof strains (Figure 2).

### 3.2. The Inactivated HOG Pathway Is the Key Location for Directed Experimental Adaptive Evolution

Expanding on our previously published observations [18], we confirmed that both types of DEA evolutionoccur only in lof-mutants with inactivated HOG pathway, namely *∆Mohik1*, *∆Moypd1*, *∆Mossk1*, *∆Mossk2*, *∆Mopbs2*, and *∆Mohog1*. Analysis of other inactivation mutants, which are osmosensitive but not related to the HOG pathway, such as (*∆Moskn7* (MGG_03516), *∆Mostu1* (MGG_04185), *∆MofluG* (MGG_16491), *∆Mompg1* (MGG_10315), *∆Mossp2* (MGG_00803), *∆Monpr2* (MGG_00664) and *∆Mopmk1* (MGG_09565), revealed that DEA evolution is strictly limited to lof mutants of the HOG pathway. We demonstrated in growth assays that no mycelium parts arose out of all non-HOG-related osmosensitive mutant strains, whereas the reversible as well as the irreversible suppressors grew out of the mutants with an inactivated HOG pathway.

**Figure 3:**
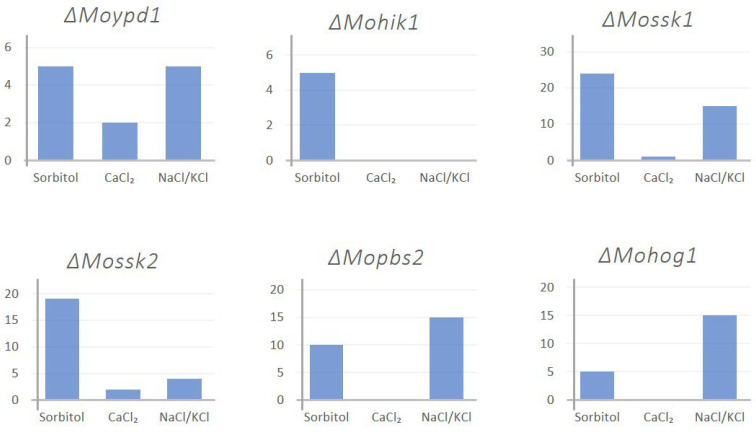
Example of empirical investigation of the DEA evolution events upon different stressors. The y-axis represents the number of generated suppressor mutants for the respective HOG inactivation mutant.

Furthermore, we added empirical investigations of different stress parameters which were known to trigger the HOG pathway, like CaCl_2_ (Ion-stress), CoCl_2_ (hypoxia), and NaNO_2_ (salt stress and hypoxia), fludioxonil (fungicide stress), temperature and light/dark rhythm in order to find different factors promoting or constraining DEA evolution (see material and methods, and [14]). Apart from osmotic stress (NaCl, KCl, sorbitol), additional agents like CaCl_2_ (Ion-stress), CoCl_2_ (hypoxia), and NaNO_2_ (salt stress and hypoxia), different pH-values, and fludioxonil (fungicide stress) were not successful in the generation of suppressor mutants. The lof-mutants ∆Mohik1, ∆Moypd1, ∆Mossk1, ∆Mossk2, ∆Mopbs2, and ∆Mohog1 have been cultivated for more than 10 weeks on medium supplemented with sublethal concentrations of stress-inducing agents previously mentioned (Figure 3).

Referring to Figure 3, sorbitol, as a strong stressor, generated a relatively high number of suppressor mutants in all HOG inactivation mutants. Similar results were observed with NaCl/KCl treatment except for ΔMohik1. On the other hand, CaCl_2_ was only able to generate a few mutants in ΔMoypd1, ΔMossk1and ΔMossk2 and none in the rest.

### 3.3. Reintegration of Originally HOG Pathway-Inactivated Genes in the Suppressor Strains Restores Naturally Biochemical and Physiological Phenotype

Since we know that the suppressor strains arose only out of lof mutants with inactivated HOG pathway, one fundamental question driving our research is, to identify the impact of a genetic reconstruction of the functional HOG pathway on the phenotypic characteristics of the suppressor strains ∆Mohik1(reversible), ∆Moypd1(reversible), ∆Mossk1(reversible), ∆Mossk2(reversible), ∆Mopbs2(reversible) and ∆Mohog1(reversible) as well as on ∆Mohik1(irreversible), ∆Moypd1(irreversible), ∆Mossk1(irreversible), ∆Mossk2(irreversible), ∆Mopbs2(irreversible) and ∆Mohog1(irreversible). Therefore, we reintegrated the original lof gene into the genome of the respective lof mutants (classical complementation experiment) and in each reversible and each irreversible suppressor strain. The resulting strains were named ∆Mohik1/HIK1, ∆Moypd1/YPD1, ∆Mossk1/SSK1, ∆Mossk2/SSK2, ∆Mopbs2/PBS2 and ∆Mohog1/HOG1, the complemented suppressors were named ∆Mohik1(reversible)/HIK1, ∆Moypd1(reversible)/YPD1, ∆Mossk1(reversible)/SSK1, ∆Mossk2(reversible)/SSK2, ∆Mopbs2(reversible)/PBS2 and ∆Mohog1(reversible)/HOG1 as well as ∆Mohik1(irreversible)/HIK1, ∆Moypd1(irreversible)/YPD1, ∆Mossk1(irreversible)/SSK1, ∆Mossk2(irreversible)/SSK2, ∆Mopbs2(irreversible)/PBS2 and ∆Mohog1(irreversible)/HOG1. We then monitored the complemented suppressors for vegetative growth upon stress, production of compatible solutes, as well as virulence, and compared them to the lof mutant and wildtype strains.

**Figure 4:**
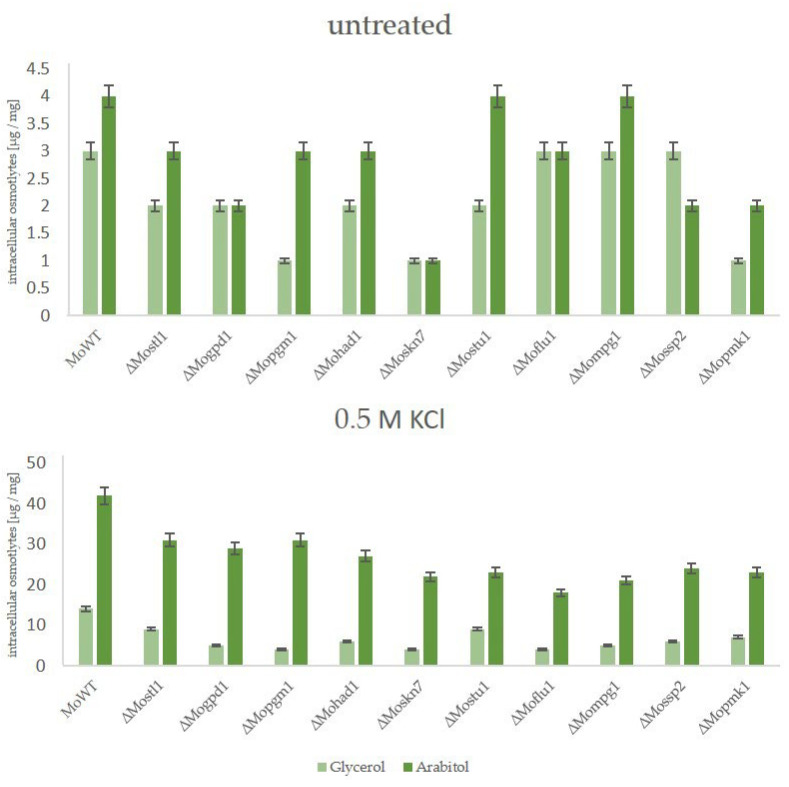
The production of compatible solutes as a stress response in the *Magnaporthe oryzae* wildtype strain (MoWT), and mutant strains with reintegrated lof-gene of the HOG pathway. The fungal strains were grown for 72 h in CM before being shocked with 0.5 M KCl. Carbohydrates were extracted after 7 h and quantified by HPAEC-PAD. Error bars represent the standard deviation of three biological replicates of each strain. Intracellular osmolyte concentration is given in µg/mg mycelial dry weight.

To begin with, intracellular production of compatible solutes was determined by HPAEC-PAD, where arabitol was clearly demonstrated to be the major component produced after stress in all the complemented strains. Arabitol production was found to be almost as high as it was detected in the wildtype strain (Figure 4).

**Figure 5:**
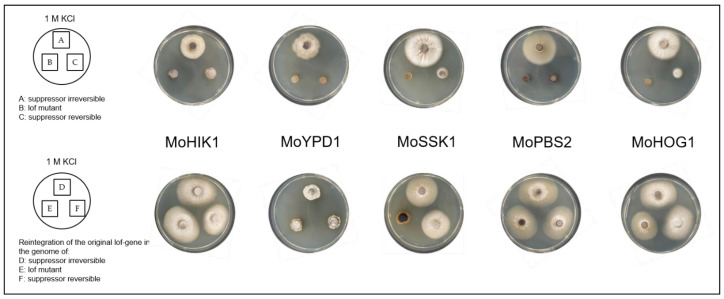
Vegetative growth of the *Magnaporthe oryzae* lof mutants, the suppressor mutants (irreversible and reversible), and the strains with the reintegration of the original lof-gene of the HOG pathway into the genome. The cultures were grown on media with osmotic stress (1 M KCl) for 7 days at 26 °C.

Furthermore, the genetic reconstruction and consequently a functional HOG pathway in the lof mutants as well as in the suppressor strains (irreversible and reversible) resulted not only in the production of arabitol as major stress response upon osmotic stress again, but also in reestablishing osmoregulation capacity in the lof mutants (except for *∆Mossk1*) as well as in all the suppressor strains (Figure 5).

**Figure 6:**
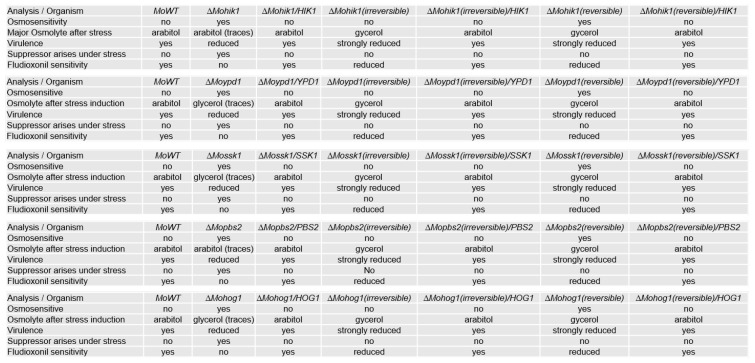
Overview of the most important phenotypes of the *Magnaporthe oryzae* lof mutants, the suppressor strains (reversible and irreversible) as well as the strains in which the HOG pathway is genetically reactivated.

Additionally, it is documented that the lof mutants with inactivated HOG pathway *∆Mohik1*, *∆Moypd1*, *∆Mossk1*, *∆Mossk2*, *∆Mopbs2*, and *∆Mohog1* are reduced in virulence as compared to the wildtype strain (Jacob et al., 2015 [13]). Interestingly, the suppressor strains were shown to be even less virulent as compared to the lof mutants [18]. In this study, we found, that virulence was not only reconstituted in the complemented original lof strains *∆Mohik1/HIK1*, *∆Moypd1/YPD1*, *∆Mossk1/SSK1*, *∆Mossk2/SSK2*, *∆Mopbs2/PBS2* and *∆Mohog1/HOG1*, but also in all the tested complemented suppressors *∆Mohik1(reversible)/HIK1*, *∆Moypd1(reversible)/YPD1*, *∆Mossk1(reversible)/SSK1*, *∆Mossk2(reversible)/SSK2*, *∆Mopbs2(reversible)/PBS2* and *∆Mohog1(reversible)/HOG1* as well as in *∆Mohik1(irreversible)/HIK1*, *∆Moypd1(irreversible)/YPD1*, *∆Mossk1(irreversible)/SSK1*, *∆Mossk2(irreversible)/SSK2*, *∆Mopbs2(irreversible)/PBS2* and *∆Mohog1 (irreversible)/HOG1* (Figure 6).

Fludioxonil susceptibility is an important characteristic feature which was found to be reestablished in the complemented suppressor strains ∆Mohik1(reversible)/HIK1, ∆Moypd1(reversible)/YPD1, ∆Mossk1(reversible)/SSK1, ∆Mossk2(reversible)/SSK2, ∆Mopbs2(reversible)/PBS2 and ∆Mohog1(reversible)/HOG1 as well as in ∆Mohik1(irreversible)/HIK1, ∆Moypd1(irreversible)/YPD1, ∆Mossk1(irreversible)/SSK1, ∆Mossk2(irreversible)/SSK2, ∆Mopbs2(irreversible)/PBS2 and ∆Mohog1(irreversible)/HOG1. Fludioxonil resistance was only observed for the lof mutants with a non-functional HOG pathway, whereas all the suppressor strains (reversible and irreversible), as well as all lof strains in which the HOG pathway was genetically reconstructed, were found to be as sensitive to the fungicide as the wildtype strain (Figure 6). Additionally, the genetically reconstructed mutants with the possibly reactivated HOG pathway are not able to generate further suppressor mutants via DEA evolution.

### 3.4. The Difference of the Biochemical Stress Response in Reversible and Irreversible Suppressor Strains

**Figure 7:**
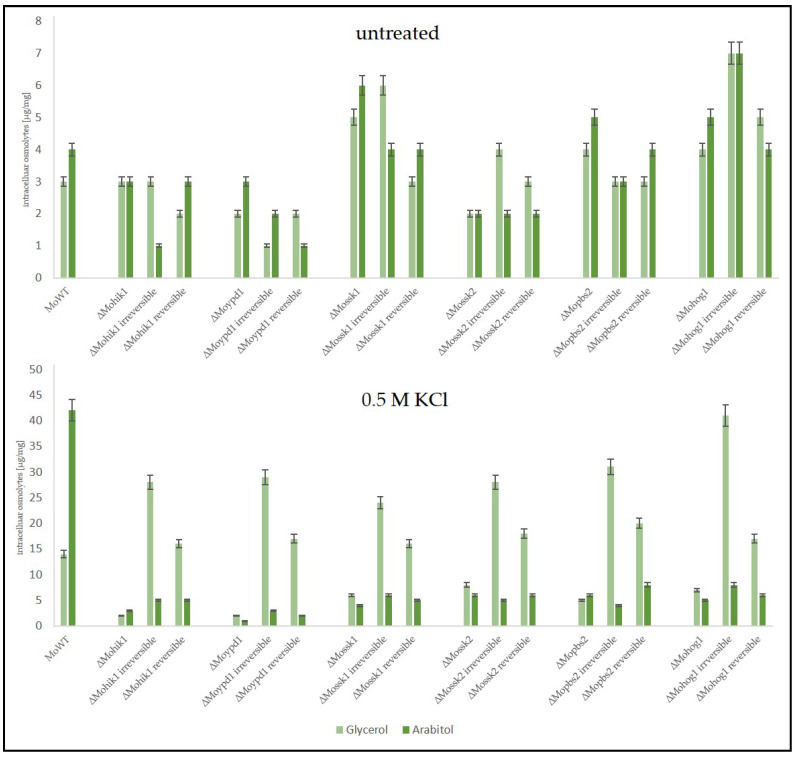
The production of compatible solutes as a stress response in the *Magnaporthe oryzae* wildtype strain (WT), the lof mutants of the HOG pathway, and the suppressor strains (irreversible and reversible). The fungal strains were grown for 72 h in CM (2% glucose) before being shocked with 0.5 M KCl. Carbohydrates were extracted 7 h after KCl application and quantified by HPAEC-PAD. Error bars represent the standard deviation of three biological replicates of each strain. Intracellular osmolyte concentration is given in µg/mg mycelial dry weight.

The ability of the suppressor mutants to cope with osmotic stress by producing glycerol as a compatible solute, in contrast to arabitol as it is in the wildtype strain, was further analyzed for both types of suppressors. Therefore, the wildtype strain, the lof mutants of the HOG pathway, ∆Mohik1(reversible), ∆Moypd1(reversible), ∆Mossk1(reversible), ∆Mossk2(reversible), ∆Mopbs2(reversible) and ∆Mohog1(reversible) as well as ∆Mohik1(irreversible), ∆Moypd1(irreversible), ∆Mossk1(irreversible), ∆Mossk2(irreversible), ∆Mopbs2(irreversible) and ∆Mohog1(irreversible) were grown upon osmotic stress followed by a HPAEC-PAD analysis of intracellular osmolyte content. As a result, a significant difference was detected for osmolyte production between reversible and irreversible strains (Figure 7).

As expected, the wildtype strain produced intracellular arabitol as a major solute in order to compensate for extracellular osmotic stress (0.5 M KCl), whereas it produced only small amounts of glycerol.

In contrast, the osmosensitive lof mutants *ΔMohog1*, *ΔMopbs2*, *ΔMossk2*, *ΔMossk1*, and *ΔMoypd1* were not able to produce either arabitol or glycerol in significant amounts after salt stress. In contrast, both types of suppressor strains, reversible and irreversible, produced glycerol as the main component in their biochemical stress response. Interestingly, the amount of glycerol was found to be strongly different between reversible and irreversible suppressors. Glycerol content in *∆Mohik1(irreversible)*, *∆Moypd1(irreversible)*, *∆Mossk1(irreversible)*, *∆Mossk2(irreversible)*, *∆Mopbs2(irreversible)* and *∆Mohog1(irreversible)* could be determined as almost twice as high as compared to *∆Mohik1(reversible)*, *∆Moypd1(reversible)*, *∆Mossk1(reversible)*, *∆Mossk2(reversible)*, *∆Mopbs2(reversible)* and *∆Mohog1(reversible)* (Figure 7).

### 3.5. Glycerol Metabolism-Associated Genes Are Not the Driving Force for DEA Evolution

Since the reversible and the irreversible suppressor mutants produce glycerol as a biochemical response in order to compensate high external osmolarity and furthermore, since we documented in a previous study [18] that glycerin metabolism (gm)-associated genes have been upregulated in a suppressor strain ΔMohog1(suppressor) upon salt stress, we now checked the relationship of genes related to the production, metabolism or transport of glycerol to the DEA evolution in the lof mutants of the HOG pathway. To do that, we inactivated putative gm-related genes. In more detail, a distinct set of gm-candidate genes was found to be upregulated in both the salt stress samples of the suppressor strain ΔMohog1(suppressor) as well as in the wildtype strain, whereas exactly these genes were not significantly regulated in the lof mutant ΔMohog1 [18]. The most prominent candidates were the genes encoding the glycerol H+-symporter MoStl1p (MGG_09852), the phosphoglycerate mutase (MGG_06642; MpPga1p), the glycerol-3-phosphate dehydrogenase (MGG_00067; MoGpd1p), and the phosphatidyl synthase (MGG_00099; MoHad1p) (see Supplementary Materials).

**Figure 8:**
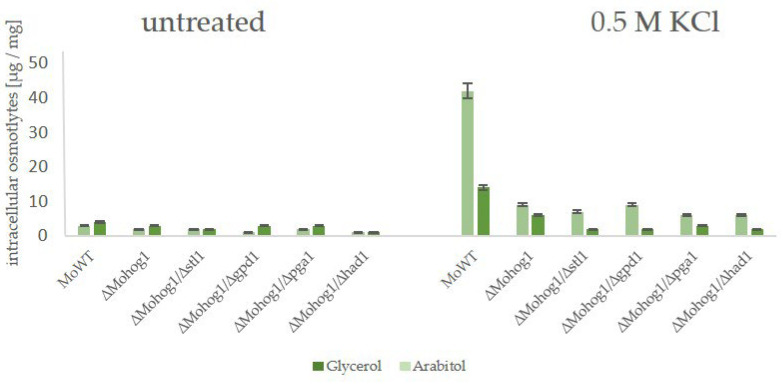
Production of compatible solutes as stress response in *Magnaporthe oryzae* wildtype strain (WT), the lof mutant ∆Mohog1, and the double mutant strains *∆Mohog1/∆stl1*, *∆Mohog1/∆pga1*, *∆Mohog1/∆gpd1*, and *∆Mohog1/∆had1*. The fungal strains were grown for 72 h in CM before being shocked with 0.5 M KCl. Carbohydrates were extracted 7 h after KCl application and quantified by HPAEC-PAD. Error bars represent the standard deviation of three biological replicates of each strain. Intracellular osmolyte concentration is given in µg/mg mycelial dry weight.

In order to evaluate whether these candidate genes have an impact on the molecular mechanism of DEA evolution in the rice blast fungus and to point out if there is a difference between the reversible and irreversible strains, we generated a set of so-called “double lof mutants”. That means, we replaced the gm-associated candidate genes MoSTL1, MoPGA1, MoGPD1, and MoHAD1 in the genomes of the wildtype strain as well as in the genome of the lof mutant ∆Mohog1 with an antibiotic resistance marker gene (see supplementary Materials). The double mutants were named ∆Mohog1/∆stl1, ∆Mohog1/∆pga1, ∆Mohog1/∆gpd1, and ∆Mohog1/∆had1. Then, we monitored whether these double-mutants were able to evolve into DEA evolution suppressor strains, as was the case for the original ∆Mohog1, checked their osmosensitivity, and analyzed their biochemical stress response by HPAEC-PAD. It could be documented that molecular-verified double mutants ∆Mohog1/∆stl1, ∆Mohog1/∆gpd1, ∆Mohog1/∆pga1, and ∆Mohog1/∆had1 are still as osmosensitive as the lof mutant ∆Mohog1. They did not produce either glycerol or arabitol in significant amounts as a stress response (Figure 8).

## 4. Discussion

Knowledge about the evolutionary dynamics in adaptation processes is still limited in eukaryotic organisms. In particular, the rapid evolutionary adaptation of signaling pathways has not been subjected to intensive research so far. One central pillar on the road to unravel evolutionary dynamics is to better understand the molecular mechanisms of how signaling pathways can be modified and rewired in order to generate altered biochemical reactions or novel physiological properties [25]. How have signaling networks evolved, and what are the molecular mechanisms behind them? It is of great interest to better understand how evolutionary changes can promote or constrain signaling networks of pathogenic microorganisms, enabling them to live beneficially within their host or their environment. Insights into the molecular basis of evolutionary dynamics can help to better understand the biology of pathogenic fungi and, as a consequence, will help to search for new disease control strategies. It is of high importance to understand when and how cross-talk between signaling pathways evolves or how rewiring of signaling pathways takes place in order to rearrange or modify them, generating, for example, antibiotic resistance (multi-drug-resistance) [8,25]. The function of the HOG pathway in fungi is to regulate cellular homeostasis and, therefore, the adaptation to rapidly changing osmolarity in the environment [15]. The lof mutants *ΔMohog1*, *ΔMopbs2*, *ΔMossk2*, *ΔMossk1*, and *ΔMoypd1* of the phytopathogenic fungus *Magnaporthe oryzae* are impaired in osmoregulation and resistant to the fungicide fludioxonil [13]. Previously, it was discovered that long-term cultivation of osmosensitive HOG pathway lof mutants upon high osmolarity for several weeks resulted in so-called suppressor strains (formerly known as “adapted” strains) being restored in osmoregulation outgrowing from each of the lof mutants *ΔMohog1*, *ΔMopbs2*, *ΔMossk2*, *ΔMossk1*, and *ΔMoypd1* [18]. However, this is completely different from processes already known as “experimental evolution”. Experimental evolution studies of evolving microbial populations (not individuals) nowadays form the fundamentals of the theory of evolution [26]. The experiments begin with a culture; the microbial cells are cultivated in liquid medium and grown to a high population density. Then, parts of the culture are transferred to a new medium, or the culture gets diluted with a fresh medium to allow continued growth. This process can be continued indefinitely, and new generations accumulate under natural selection [27]. The most popular and longest-running experimental evolution experiment is the “long-term evolution experiment” (LTEE) [27,28]. Twelve replicate populations of *E. coli* have been cultivated continuously since 1987 and have reached over 68,000 generations. All these evolution experiments use “populations” whereas in this report, we describe a rapid evolutionary suppressor phenomenon that is extremely reproducible in single individuals with a non-functional HOG pathway growing on solid medium upon high osmolarity. Interestingly, the major compatible solute produced by the *M. oryzae* suppressor strains to cope with osmotic stress was glycerol, whereas it is arabitol in the wildtype strain. By studying the rapidly evolved suppressor strains in more detail, it was found that the suppression is at least a two-step process. In step one, suppressor strains with temporarily (reversible) reestablished osmoregulation arise from the osmosensitive lof-mutants upon several weeks of cultivation on permanent osmotic stress. Further maintenance of these suppressor strains on high osmolarity led to a second dynamic step of rapid adaptation. The reversible suppressor strains then transform into irreversible strains able to cope with high osmolarity due to a permanently reestablished osmoregulation (Figure 1). It could be clearly demonstrated for the first time that the course of rapid evolution of the osmoregulation in *M. oryzae* is a highly reproducible process that takes place in several steps, making it very suitable for the investigation of evolutionary dynamics. While Adaptive Laboratory Evolution (ALE) stands as a valuable tool in elucidating evolutionary dynamics, its application may not be suitable in the context described. The phenomenon under investigation involves the emergence of suppressor strains from osmosensitive mutants of the high osmolarity glycerol (HOG) pathway in *M. oryzae*, with a notable shift in osmoregulatory responses favoring glycerol over arabitol production. In this scenario, the iterative process of ALE, which involves subjecting microbial populations to specific environmental stresses over multiple generations, may not offer substantial insight. The ability to replicate the observed phenomenon with different mutants and on various media suggests that the emergence of suppressor strains is reproducible under controlled laboratory conditions without the need for prolonged evolutionary experiments facilitated by DEA evolution. Instead, the focus may shift toward targeted genetic and physiological analyses to unravel the underlying mechanisms driving the observed adaptations. In order to address this phenomenon, biochemical analysis, genetic manipulation, and proteome- as well as phospho-proteome-profiling was performed.

A reintegration of the original lof-genes in the genomes of the suppressor mutants resulted in complemented strains producing arabitol as the main compatible solute upon osmotic stress, in contrast to glycerol in the suppressor strains. This leads to the assumption that a functional HOG pathway is preferred by the organism as the osmoregulatory system, in contrast to the “new” evolved suppression mechanism. In the era of next-generation sequencing, bioinformatics has become a major discipline in biology for analyzing evolutionary processes [29]. In order to study the putative role in evolutionary dynamics of the genes which were found to be upregulated in NGS datasets of the reversible and irreversible suppressor strains [18], lof mutants of the genes encoding the glycerol H+-symporter MoStl1p (MGG_09852), the phosphoglycerate mutase (MGG_06642; MpPga1p), the glycerol-3-phosphate dehydrogenase (MGG_00067; MoGpd1p) and the phosphatidyl synthase (MGG_00099; MoHad1p) have been generated in the lof mutant ∆Mohog1 and characterized.

We observed that these candidate genes were not involved in the two-step process of reversible and irreversible suppression, since the double mutant strains ∆Mohog1/∆stl1, *∆Mohog1/∆gpd1*, *∆Mohog1/∆pga1*, and *∆Mohog1/∆had1* were still able to transform to suppressors upon permanent osmotic stress. In order to generate glycerol, the NADP+ dependent glycerol dehydrogenase is required and not the glycerol 3-phosphate dehydrogenase. Even though the possible candidate has now been noticeable in the different analyses so far, the NADP+ dependent glycerol dehydrogenase should be the focus of further research in order to find the possible connection between the metabolic switch. The Mitochondrial glycerol-3-phosphate shuttle consists of two components: a cytoplasmic glycerol-3-phosphate dehydrogenase 1 (MoGdp1_MGG00067) and a mitochondrial glycerol-3-phosphate dehydrogenase 2 (MoGdp2, MGG_03147). The inactivation mutant of the mitochondrial glycerol 3-phosphate dehydrogenase could not efficiently utilize glycerol as a carbon source, suggesting the involvement of GPD2 in glycerol utilization in *M. oryzae* [30,31]. In the end, we have achieved significant progress in the characterization of the suppressor phenome in *M. oryzae*. We were able to clearly demonstrate for the first time that this rapid evolution is at least a two-step process, showing the differences between reversible and irreversible suppressor strains. This provides a strong basis for future studies to elucidate the exact molecular mechanism behind this dynamic evolution.

## 5. Conclusions

In this study, we demonstrate that directed experimental adaptive (DEA) evolution in the rice blast fungus *Magnaporthe oryzae* is a highly reproducible and stepwise process that occurs specifically in loss-of-function mutants of the HOG pathway. Two distinct suppressor types, reversible and irreversible, emerged under long-term osmotic stress, each characterized by different capacities to restore osmoregulation and by a metabolic shift toward glycerol as the primary compatible solute. Reintegration of functional HOG pathway genes restored the original physiological phenotype, emphasizing the central role of this signaling cascade in fungal stress adaptation. Importantly, inactivation of glycerol metabolism-associated genes did not prevent the formation of suppressor strains, indicating that DEA evolution operates independently of canonical glycerol metabolic routes.

These findings provide a framework for understanding rapid adaptive processes in filamentous fungi and highlight the HOG pathway as a model for investigating evolutionary processes in signaling networks. Future work should aim to elucidate the molecular determinants driving this adaptive switch, including the possible involvement of alternative redox-dependent enzymes and post-translational regulation mechanisms, to better understand the dynamics of stress evolution in pathogenic fungi.

## Data Availability

All the data supporting the reported results are found in the manuscript or the Supporting Material.

## Supplementary Materials

The following supporting information can be downloaded at https://www.mdpi.com/article/10.3390/biology14111545/s1, Figure S1: Southern blot analysis and schematic presentation of the transformation process by homologous recombination and verification of the inactivation mutants, suppressor mutants (irreversible and reversible) within the *Magnaporthe oryzae* genome; Schematic representation of the genomic DNA of the *Magnaporthe oryzae* HOG inactivation mutants, suppressor mutants (irreversible and reversible) and the mutants with the reintegration of the inactivated gene with the different hybridization sizes; Figure S2: Schematic representation of the genomic DNA of the *Magnaporthe oryzae* HOG inactivation mutants, suppressor mutants (irreversible and reversible) and the mutants with the reintegration of the inactivated gene with the different hybridization sizes; Figure S3: Schematic representation of the genomic DNA of the *Magnaporthe oryzae* HOG inactivation mutants, suppressor mutants (irreversible and reversible) and the mutants with the reintegration of the inactivated gene with the different hybridization sizes; Figure S4: Schematic representation of the genomic DNA of the *Magnaporthe oryzae* HOG inactivation mutants, suppressor mutants (irreversible and reversible) and the mutants with the reintegration of the inactivated gene with the different hybridization sizes.
