# Supplementary material for: Directed Experimental Adaptive Evolution of Osmoregulation in Fungal Pathogen Magnaporthe oryzae Is Independent of Glycerol Metabolism-Associated Genes
Source: Biology (Basel). 2025 Nov 4;14(11):1545. doi: 10.3390/biology14111545 (PMC12650440; doi:10.3390/biology14111545)

Supplementary Figure S1

Southern blot analysis and schematic presentation of the transformation process by homologous recombination and verification of the inactivation mutants, suppressor mutants (irreversible and reversible) within the *Magnaporthe oryzae* genome. The genomic DNA of the different mutants (inactivation mutants, suppressor mutants (irreversible and reversible)) was isolated and restricted by restriction enzymes.

Schematic representation of the genomic DNA of the *Magnaporthe oryzae* HOG inactivation mutants, suppressor mutants (irreversible and reversible) and the mutants with the re-integration of the inactivated gene with the different hybridization sizes. The sizes next to the images of the X-ray films indicate the respective fragment size of the hybridization signals.

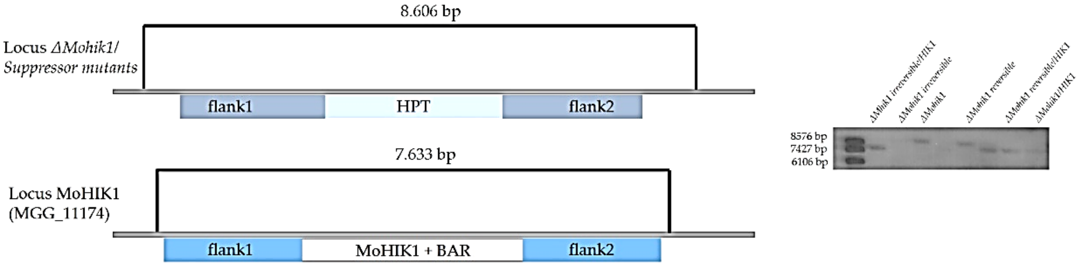

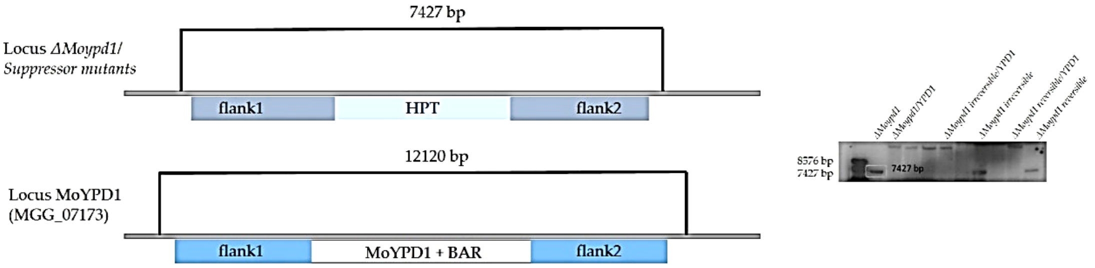

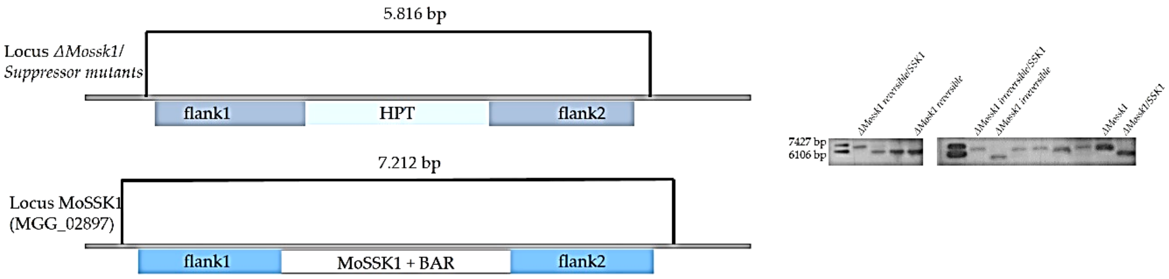

Supplementary Figure S2

Schematic representation of the genomic DNA of the *Magnaporthe oryzae* HOG inactivation mutants, suppressor mutants (irreversible and reversible) and the mutants with the re-integration of the inactivated gene with the different hybridization sizes. The sizes next to the images of the X-ray films indicate the respective fragment size of the hybridization signals.

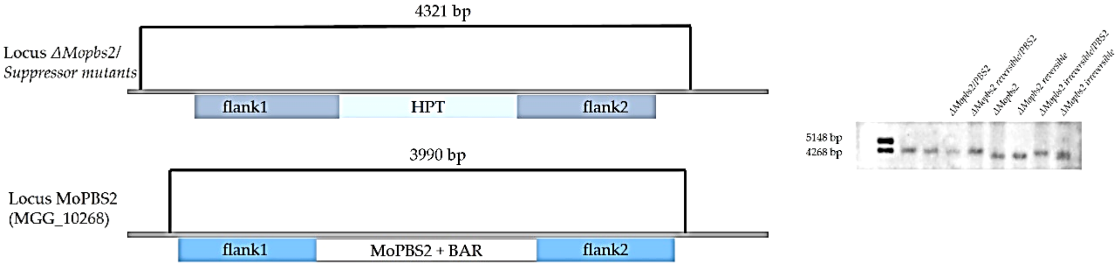

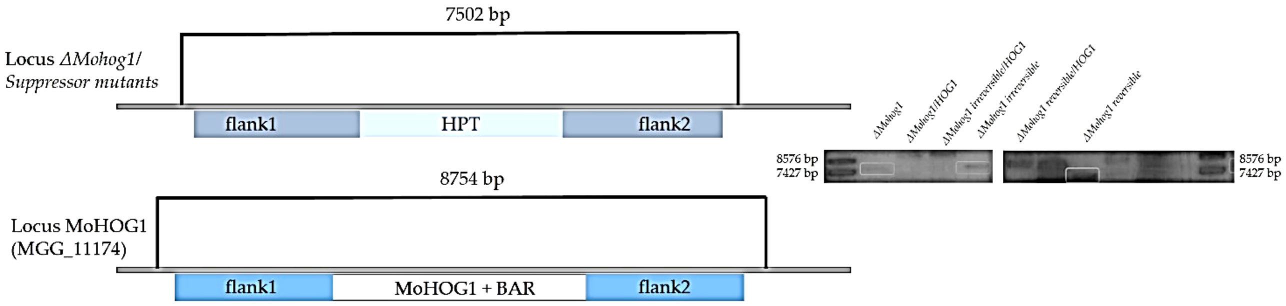

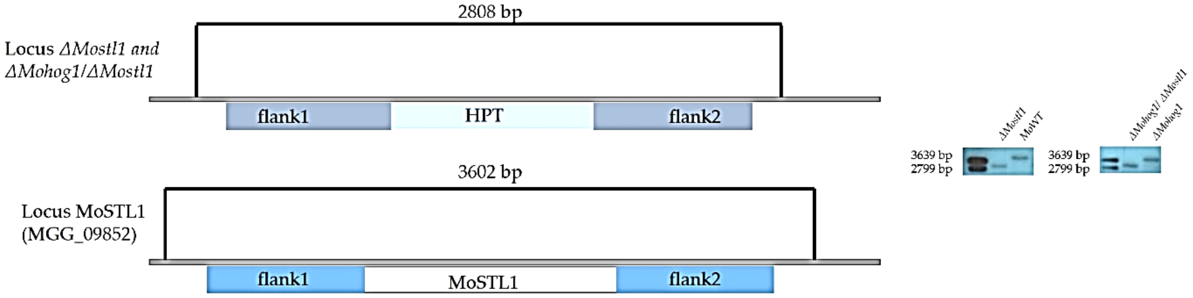

Supplementary Figure S3

Schematic representation of the genomic DNA of the *Magnaporthe oryzae* HOG inactivation mutants, suppressor mutants (irreversible and reversible) and the mutants with the re-integration of the inactivated gene with the different hybridization sizes. The sizes next to the images of the X-ray films indicate the respective fragment size of the hybridization signals.

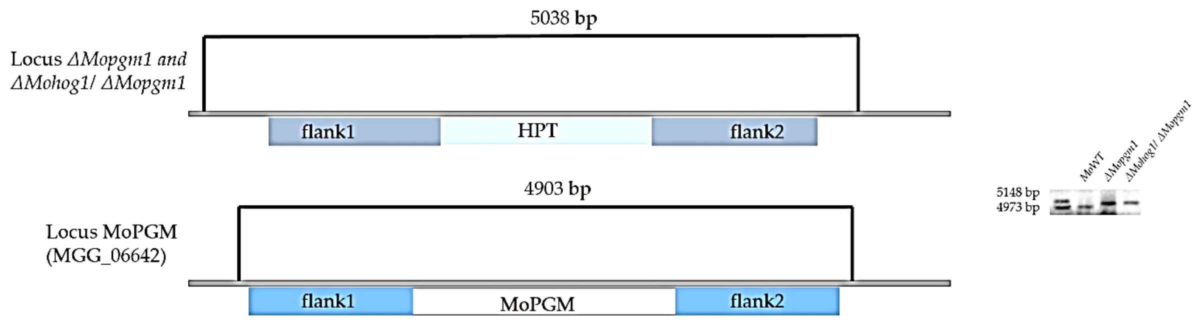

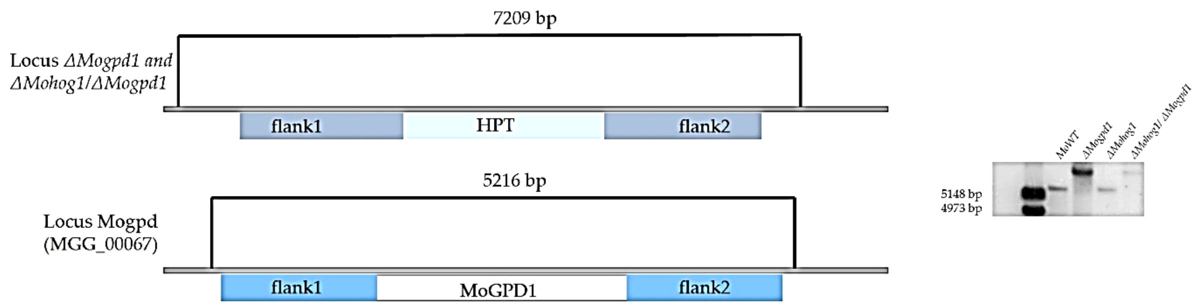

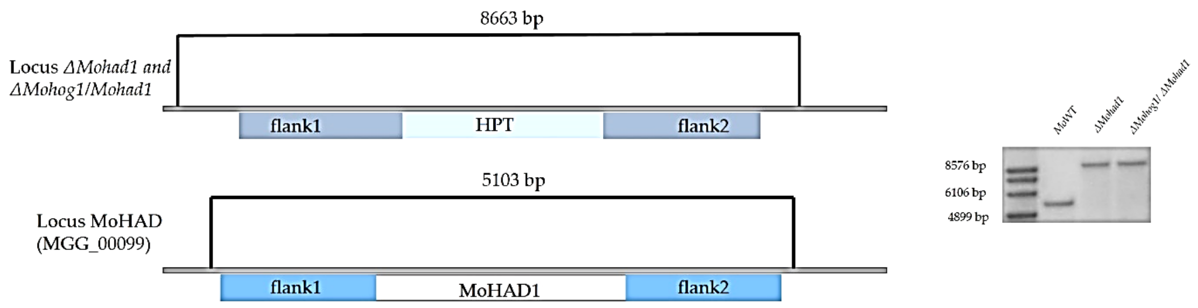

Supplementary Figure S4

Schematic representation of the genomic DNA of the *Magnaporthe oryzae* HOG inactivation mutants, suppressor mutants (irreversible and reversible) and the mutants with the re-integration of the inactivated gene with the different hybridization sizes. The sizes next to the images of the X-ray films indicate the respective fragment size of the hybridization signals.

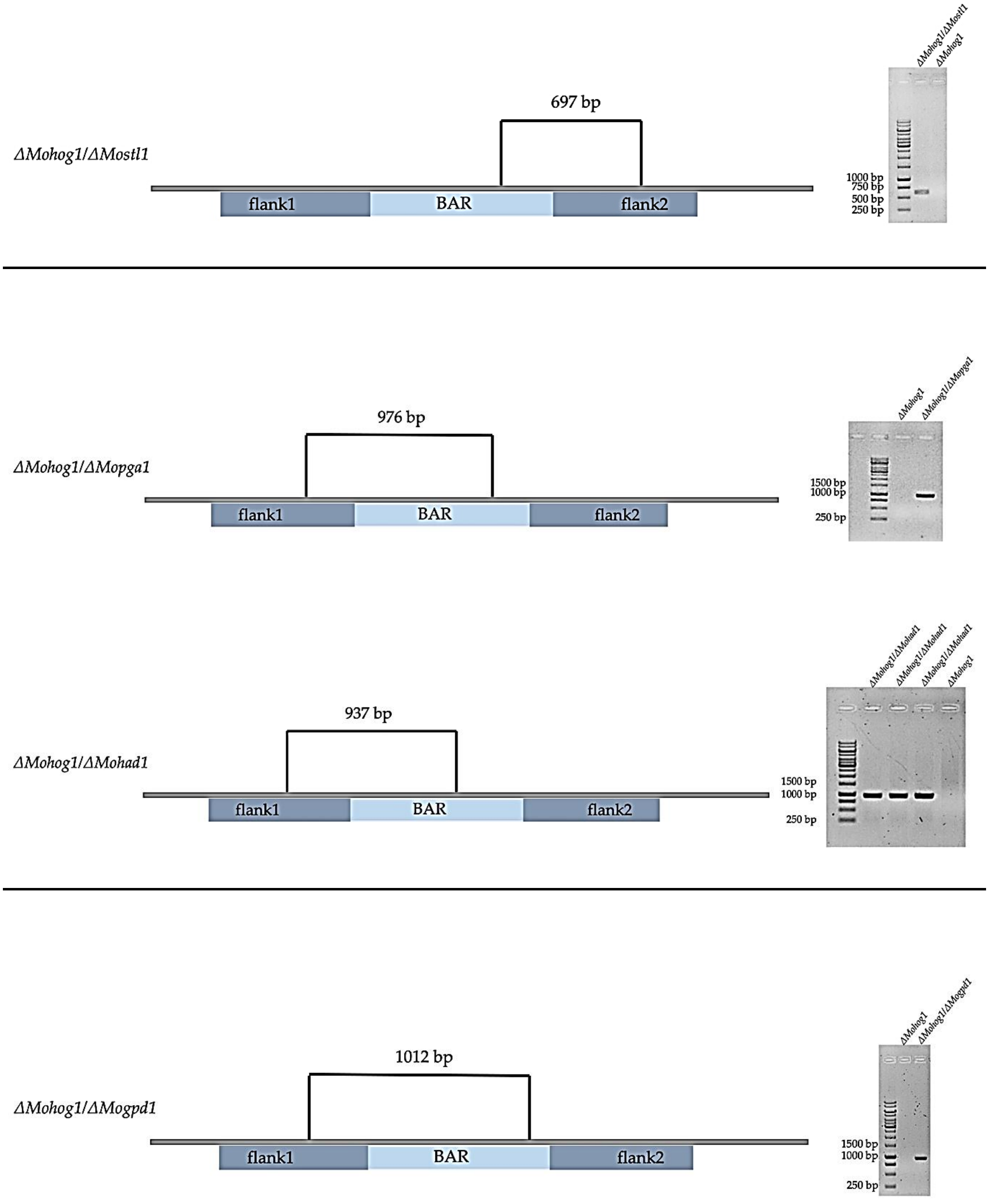

Supplement: Supplementary file 1 [file biology-14-01545-s001.zip › biology-3912565-supplementary.pdf]
